# ﻿First country record of *Metopiellus* Raffray, 1908 (Staphylinidae, Pselaphinae, Metopiasini) from Ecuador, with description of two new species

**DOI:** 10.3897/zookeys.1266.169869

**Published:** 2026-01-08

**Authors:** Yarina Tapuy-Avilés, David R. Díaz-Guevara, Michael S. Caterino

**Affiliations:** 1 Pontificia Universidad Católica de Ecuador, Quito, Ecuador Fundación Uru Quito Ecuador; 2 Fundación Uru, Quito, Ecuador Pontificia Universidad Católica de Ecuador Quito Ecuador; 3 Instituto Nacional de Biodiversidad, Quito, Ecuador Instituto Nacional de Biodiversidad Quito Ecuador; 4 Clemson University, Clemson, South Carolina, USA Clemson University Clemson United States of America

**Keywords:** Ant-like litter beetles, identification key, myrmecophily, new record, pselaphine beetles, taxonomy, tropical biodiversity

## Abstract

Two new species from the genus *Metopiellus* Raffray, 1908 (Staphylinidae, Pselaphinae, Metopiasini) are described from both sides of the Ecuadorian Andes: *Metopiellus palamaku***sp. nov.** from the Amazon in eastern Ecuador and *Metopiellus chasqui***sp. nov.** from montane cloud forest in western Ecuador. These findings represent the first country record of the genus.

## ﻿Introduction

Metopiasini is a tribe of Pselaphinae rove beetles suspected to be myrmecophiles (Parker 2016). This tribe comprises nine genera, eight of which are restricted to the Neotropical region (Mário Chaul and Lopes-Andrade 2024). One of these Neotropical genera is *Metopiellus* Raffray, 1908, which is distributed from Colombia to Argentina (Fiorentino et al. 2022; Mário Chaul and Lopes-Andrade 2024). This genus is composed of seven described species, five of them (*M. aglenus* Reitter, 1885, *M. hirtus* Reitter, 1885, *M. painensis*Asenjo et al., 2017, *M. crypticus*Asenjo et al., 2023, *M. emavieirae* Mário Chaul & Lopes-Andrade, 2024) from Brazil, one from Colombia (*M. guanano* Fiorentino, Tocora & Ramirez, 2022), and a final one from Argentina (*Metopiellus silvaticus* Bruch, 1933). *Metopiellus* is distinguished by a second antennomere much longer than the third, protibia carinated anteromedially, metacoxae contiguous or nearly so, and pronotum without sharp spines on the lateral edge, at most having thickened spinose protuberances mediolaterally on the pronotal disc (Mário Chaul and Lopes-Andrade 2024).

Until now, *Metopiellus* has not been reported from Ecuador. Herein, we officially describe two species of this genus, from both sides of the Andes: *M. palamaku* sp. nov. from an Amazon ecosystem in eastern Ecuador and *M. chasqui* sp. nov. from a montane cloud forest in western Ecuador.

## ﻿Material and methods

Some specimens of the new species were obtained by sifting forest-floor leaf litter from the Minga Reserve in Napo Province, Ecuador (0.88853°S, 77.26833°W) and from the Otongachi Reserve in Pichincha Province, Ecuador (0.330510°S, 78.934420°W). One was hand-collected at Tiputini Biodiversity Station (0.6376°S, 76.1499°W) under permit FAUNA (X) 0020-MA-DPO-PNY (Orellana Province). Sifted litter was processed using Winkler extractors, with specimens collected, stored in 95% EtOH, and finally deposited in the National Biodiversity Institute collection (MECN) and the Pontificia Universidad Católica del Ecuador (QCAZ). Genitalia were cleared using 10% KOH, rinsed in water, and stored in glycerin in microvials pinned beneath the specimens. Photos were taken with a Macropod Pro photographic system (Macroscopic Solutions LLC), using a Canon MP-E 1–5X macro lens, combining the resulting stacks with Zerene Stacker (Richland, WA, USA).

Species recognition was based on unique combinations of external and male genitalic characters. The abbreviations used in the text and figures are: **AL**—abdomen length, **BL**—body length (from the anterior margin of the prolongation of the head to the posterior margin of tergite VIII), **BW**—body width (maximum width of elytra in dorsal view), **EL**—elytral length (maximum in dorsal view), **EW**—elytral width (maximum in dorsal view), **HL**—head length (from the anterior margin of the prolongation of the head to the posterior margin of the head disc in dorsal view), **HW**—head width (maximum, including eyes, in dorsal view), **NW**—neck width (minimum in dorsal view), **PL**—pronotum length (maximum in dorsal view), **PW**—pronotum width (maximum in dorsal view).

## ﻿Results

### ﻿Key to species of *Metopiellus*

Note: Adapted from Asenjo et al. (2017), Fiorentino et al. (2022), and Mário Chaul and Lopes-Andrade (2024). See those references for additional images and illustrations.

**Table:** 

1	Vertex with projections; pronotum with two pairs of mediolateral thick, spinose projections, either simply convex or with a series of well-defined tumuli	**2**
–	Vertex without projections	**5**
2	Vertex with one horn-like projection	**3**
–	Vertex with a pair of horn-like projections	**4**
3	Pronotum with one pair of mediolateral, thick, spinose projections (Fig. 1A); each elytron with two basal foveae (Fig. 1A)	***M. palamaku* sp. nov.**
–	Pronotum with two pairs of mediolateral, thick, spinose projections; basal elytral fovea absent	***M. guanano* Fiorentino et al., 2022**
4	Vertexal projections horn-like, apically acute; pronotum with two pairs of mediolateral, thick, spinose projections and one lateral process on each side; sternite VI with a pair of mediolateral setose processes and tergite VIII with a medial subquadrate thick process in the male	***M. emavieirae* Mário Chaul & Lopes-Andrade, 2024**
–	Vertexal projections forming transverse pair of ridge-like projections (Fig. 1F); pronotum with single pair of obliquely transverse mediolateral ridge-like processes, the inner corner of each with acute point in the lateral pronotal one (Fig. 1E), in addition to acute lateral process on each side; male sternite VI and tergite VIII lacking secondary modifications	***M. chasqui* sp. nov.**
5	Head similar in width to pronotum; eyes absent	***M. aglenus* (Reitter, 1885)**
–	Head narrower than pronotum; eyes small, but conspicuous or very reduced, inconspicuous	**6**
6	Antennomere II about half as long as scape; antennomere V almost as long as antennomeres III and IV together	**7**
–	Antennomere II less than half as long as scape; antennomere V shorter than the length of antennomeres III and IV together	**8**
7	Antennomere VII rounded; dorsolateral margins strongly constricted anterad the eyes; paramere with apex bifurcated; median lobe curved and edge with long line of small teeth	***M. crypticus* Asenjo et al., 2023**
–	Antennomere VII rectangular; dorsolateral margins of head gradually converging anterad the eyes; paramere not bifurcated apically; median lobe almost straight, with the apex curved, without small teeth along its length	***M. painensis* Asenjo et al., 2017**
8	Antennomere VIII transverse; eyes small, but conspicuous	***M. hirtus* (Reitter, 1885)**
–	Antennomere VIII obconical; eyes very reduced, inconspicuous	***M. silvaticus* Bruch, 1933**

### ﻿Taxonomy

#### ﻿ Metopiellus palamaku sp. nov.

Taxon classificationAnimaliaColeopteraStaphylinidae

ECADB036-E18D-5186-866F-C8C250313EE4

https://zoobank.org/F67D4393-8C7F-4839-B576-FFC41B318BDD

Figs 1A–C, 2A–C

##### Type material.

***Holotype*** ♂ (MECN-EN 23780): “Ecuador. Napo: Chontapunta, R. Minga, -0.88692, -77.27049, 300 m. Winkler. 07-feb-2023, Díaz-Guevara & N. Berrazueta” / “MECN-EN 23780”; deposited in MECN.

##### Additional, non-type material.

Ecuador • 1 ♀; Orellana; 0.6376°S, 76.1499°W; Tiputini Biodiv. Sta.; 2-9.vi.2011; general hand collecting, AT1341, M. Caterino, A. Tishechkin; Caterino DNA voucher, Ext. MSC-12663; ZSFQ-i16787 • 1 ♀; Panayacu, Yasuní, 0°42'17.6"S, 76°40'15.7"W; 340 m; Winkler; 17 Jul. 2021; Pazmiño-Palomino; MECN-EN 48730.

##### Comparative diagnosis.

*Metopiellus palamaku* sp. nov. strongly resembles *M. guanano*, having a vertex with a single horn-like projection. However, *M. palamaku* can be easily differentiated by its single pair of mediolateral, thick, spinose pronotal projections (vs. two pairs in *M. guanano*), the presence of basal elytral foveae (absent from *M. guanano*), and by aedeagal characters including a more elongate basal bulb, and differently shaped articulated armature (Fig. 2), bifid at the tip with subapical hook bent at ~90° (vs. aedeagal armature simply tapered, slightly sinuate at apex in *M. guanano*; aedeagal armature with four spatulate, curved tips in *M. emavieirae*).

##### Description.

**Figure 1.**
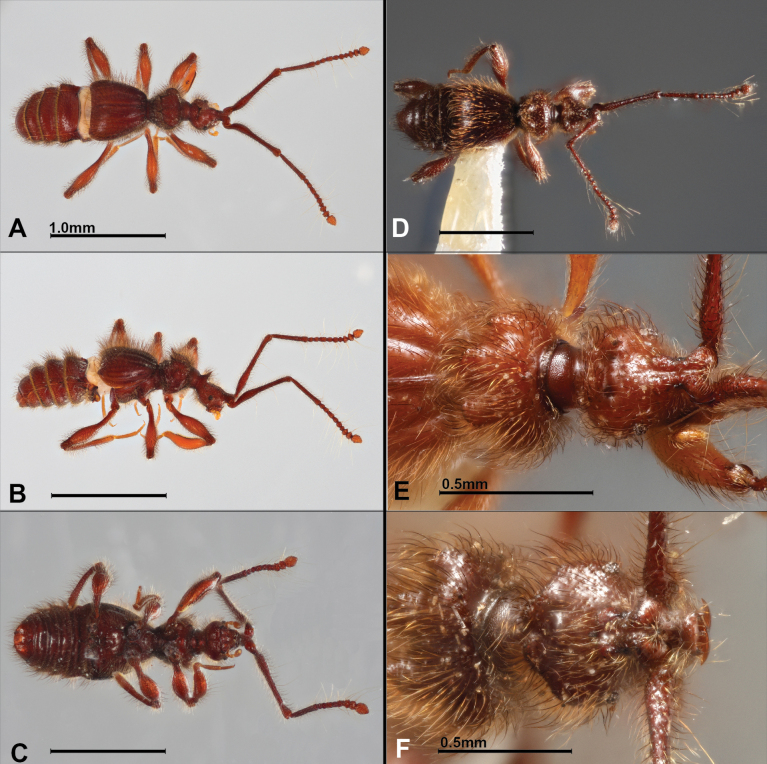
Habitus of new *Metopiellus* species. **A–C.***M. palamaku* sp. nov. (female dorsal, female lateral, male holotype ventral); **D–F.***M. chasqui* sp. nov. (male holotype dorsal, female paratype head + pronotum, male non-type head only). Scale bars: 1 mm (unless otherwise indicated).

**Figure 2.**
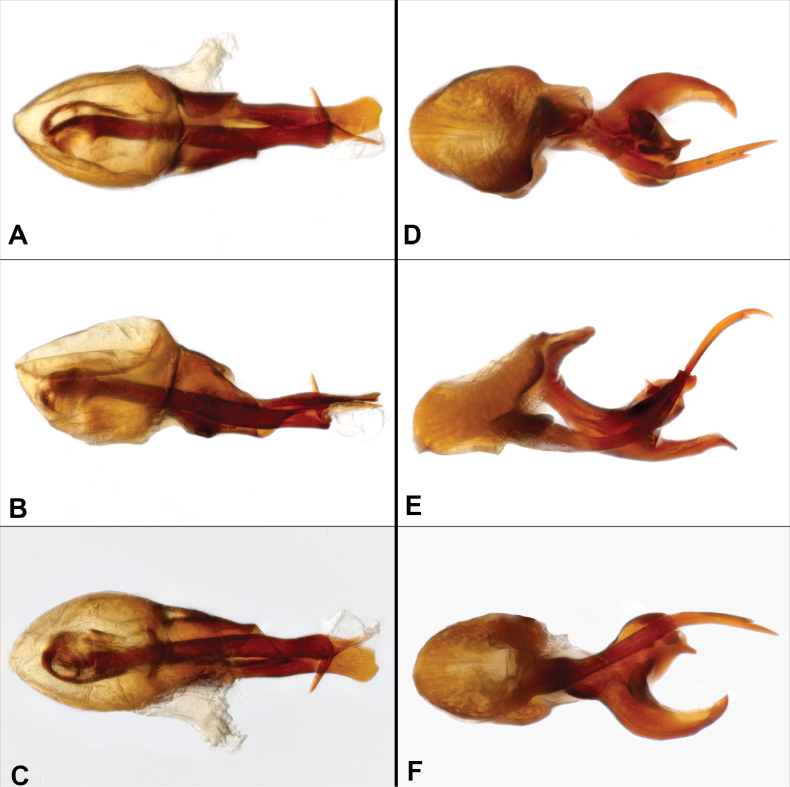
Aedeagus of new *Metopiellus* species (dorsal, lateral, ventral). **A–C.***M. palamaku* sp. nov.; **D–F.***M. chasqui* sp. nov.

Avg. BL = 2.7 mm (*N* = 2; see Table 1 for other measurements). Body (Fig. 1A–C) densely setose and brown; eyes prominent, comprising ~11 ommatidia; head pyriform with two vertexal foveae near posterior margin with a single median process similar to *M. guanano*; antennae with 11 antennomeres, antennal scape base thick and slightly narrowed before the apex (in dorsal view), antennomere I as long as rest of the antennomeres combined, antennomere II about as long as III–VIII combined, III-IV and VI slightly longer than wide, V distinctly longer, VII globular, VIII small, IX and X slightly transverse, XI almost as long as IX–X combined, tapered in apical half; pronotum trapezoidal, wider anteriorly and narrower posteriorly, with two pairs of mediolateral, thick, spinose projections; each elytron with two basal foveae, and with longitudinal impressions extending from them posteriorly approximately two-thirds elytron length. Aedeagus (Fig. 2A–C) with large, elongate basal bulb, slightly asymmetrical, with smoothly rounded basal margin; dorsal diaphragm large, elongate oval; tegmen narrowed toward flat, paddle-shaped apex; elongate armature articulated within basal bulb, apex bifid, with acute, subapical hook bent at ~90°, other tip slightly diverging to left, acute.

**Table 1.** Measurements of all species, in millimeters (mm).

Species	*N*	AL	BL	BW	EL	EW	HL	HW	NW	PL	PW
*M. palamaku* (♂)	1	1.75	2.91	1.00	0.86	1.00	0.58	0.55	0.26	0.58	0.64
*M. palamaku* (♀)	1	1.72	2.52	0.98	0.83	0.98	0.58	0.49	0.30	0.63	0.67
*M. chasqui* (♂)	1	2.16	3.24	1.07	0.94	1.07	0.56	0.56	0.25	0.52	0.70
*M. chasqui* (♀)	1	2.02	3.24	1.03	0.96	1.03	0.64	0.56	0.24	0.58	0.67

##### Distribution.

**Figure 3.**
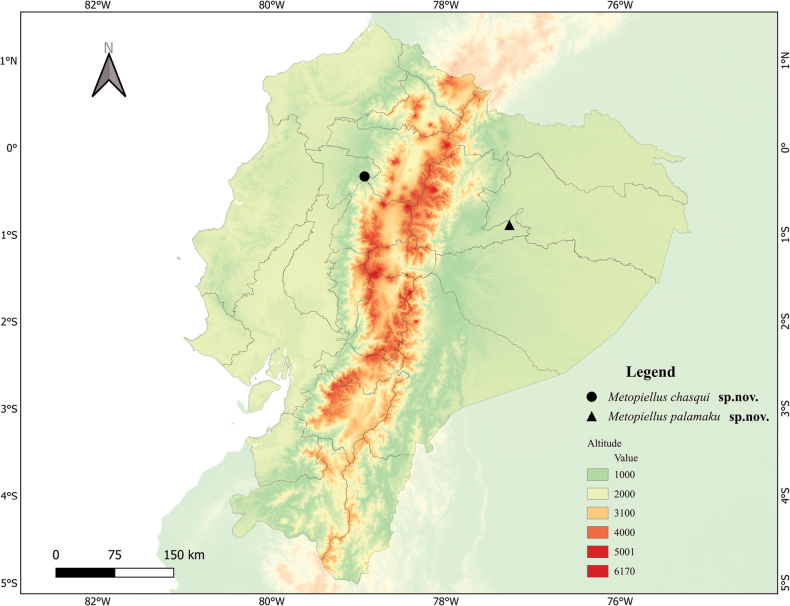
Map of Ecuador showing type localities of the two new species.

This species is known from the type locality in Chontapunta, Ecuador (Fig. 3), and probably also from the Tiputini region of Orellana (if the associations of the females under ‘other material’ are correct). The sites are separated by about 125 km, but are all on the same (south) side of the Rio Napo.

##### Etymology.

The specific name derives from a Kichwa legend in which Palamaku is the divine source of insects.

#### ﻿ Metopiellus chasqui sp. nov.

Taxon classificationAnimaliaColeopteraStaphylinidae

FABB52E7-EA8A-580E-8CCD-D2A5995C0AFA

https://zoobank.org/636C2C0D-06BF-4B70-A4C3-4B1DA9D2F850

Figs 1D–F, 2D–F

##### Type material.

***Holotype*** ♂ (QCAZ 280376): “Ecuador. Pichincha: Otongachi Reserve -0.330510, -78.934420, 1087m.20-Feb-2020, E. Tapia & N. Dupérré”; deposited in QCAZ. ***Paratypes*** (4 ♀, same general locality as type): -0.330510, -78.934420, 1087 m (QCAZ 280377 to 280380).

##### Additional, non-type material.

Ecuador • 4 ♂, 4 ♀: Zamora Chinchipe; Las Orquídeas; -4.2482, -78.6595; 877 m; E. Tapia; Sifting litter; 29-Jul-2024; QCAZ 280381 to 280388.

##### Comparative diagnosis.

*Metopiellus chasqui* resembles *M. emavieirae* in the presence of a pair of (rather than a single) vertexal structures and by having two pairs of mediolateral pronotal projections. However, it can be distinguished from it by the vertexal and median pronotal processes being transversely ridge-like, and by the lateral pronotal processes having a posterior acute point (vs. horn-like vertexal and pronotal structures in *M. emavieirae*). The aedeagi differ strongly, with that of *M. chasqui* having a strongly bifurcate dorsal armature, and that of *M. emavieirae* being subdivided subapically into four curved, spatulate tips.

##### Description.

Avg. BL = 3.2 mm (*N* = 2; see Table 1 for other measurements). Body (Fig. 1D–F) densely setose and brown-reddish; eyes prominent, borne on small elevated base, comprising ~8 ommatidia; head widest at base, rounded, narrowed anterad, with two posterior vertexal foveae (Fig. 1E) immediately in front of two low posterior vertexal ridges; antennae with 11 antennomeres, antennal scape about as long as antennomeres II–VIII combined, antennomere II as long as III–VII combined, III–VI elongate, V longer than others, VII globular, VIII smallest, IX–XI forming weak club, IX and X transverse, XI almost as long as IX and X combined, tapered in apical two-thirds; pronotum densely setose, with 4 acute lobes, lateral lobes each with posterior acute point, median ones forming oblique ridges, prominent at their inner corners; each elytron with two basal foveae, and with longitudinal impressions extending from them posteriorly approximately two-thirds elytron length; protibia rather slender. Aedeagus (Fig. 2D–F) with basal bulb asymmetrical and globose, with basal rounded margin, elevated distad, dorsal diaphragm elongate oval; tegmen with ventral portion long, slender, curved dorsad and then distad apically; articulated armature robust, deeply bifurcated, one bifurcation is hook-like, longer and evenly tapered, other more robust with small projection oriented towards distal apex of basal bulb.

##### Distribution.

This species probably only occurs in the Otongachi Reserve in northern Ecuador, in the province of Pichincha (Fig. 3). We believe the ‘additional material’ cited from Zamora-Chinchipe to be mislabeled, since that locality is over 400 km to the south, and located on the Amazonian side of the Andean crest, an unlikely distribution for a flightless beetle species. The male genitalia is identical between the two alleged localities.

##### Etymology.

The specific epithet “chasqui” refers to the agile messengers of the Inca Empire, evoking the beetle’s slender posterior legs.

## ﻿Discussion

*Metopiellus* now contains nine known species. These two new species extend the geographic range of the genus into Ecuador. The closest previous record was from Amazonian Colombia, while the bulk of the species are known from eastern and southeastern Brazil. Given the existence of the Colombian species (*M. guanano*), a record of the genus from the Ecuadorean Amazon shouldn’t be too surprising. However, the new species from the cloud forests of the western slope of the Andes represents an important extension not only geographically but also ecologically. Clearly, further sampling of leaf-litter habitats will result in continued discovery of new species across the Neotropical region.

Mário Chaul and Lopes-Andrade (2024) considered their *M. emavieirae* to be closely related to *M. guanano*, but our *M. palamaku* is even closer to *M. guanano*, differing almost exclusively in fairly minor aedeagal characters. So if species groups are to be delineated, the three should be grouped together. *Metopiellus chasqui* also shares the horn-like projections of the vertex that these three do, and would appear to be closely related as well, although its aedeagus is radically different from any of the others. Of these four, only *M. emavieirae* exhibits any significant male secondary sexual characters, with projections of the terminal tergite and sternites.

We have little to contribute to speculation on the general natural history of species in the genus, with all our specimens coming from fairly generic leaf-litter samples. Early records of some species associated with ants’ colonies have not been repeated, and the collection of one *Metopiellus* species (*M. painensis*; Asenjo et al. 2017) from caves suggests something rather different: that members of the genus may have subterranean propensities, habits that would be consistent with their generally reduced eyes and elongated appendages. We can only hope that future direct collections take closer note of more significant natural history associations to further illuminate the biology of these impressive pselaphine beetles.

## Supplementary Material

XML Treatment for Metopiellus palamaku

XML Treatment for Metopiellus chasqui

## ﻿Additional information

### Use of AI

No use of AI was reported.

### Author ORCIDs

Yarina Tapuy-Avilés https://orcid.org/0009-0001-2357-7916

David R. Díaz-Guevara https://orcid.org/0009-0001-4632-4460

Michael S. Caterino https://orcid.org/0000-0002-2597-5707

### Data availability

All of the data that support the findings of this study are available in the main text.
